# Development of Biofidelic Skin Simulants Based on Fresh Cadaveric Skin Tests

**DOI:** 10.3390/ebj5040040

**Published:** 2024-12-16

**Authors:** Gurpreet Singh, Pramod Yadav, Arnab Chanda

**Affiliations:** 1 Centre for Biomedical Engineering, Indian Institute of Technology Delhi, New Delhi 110016, India; gurpreet.singh@cbme.iitd.ac.in (G.S.); bmz248425@iitd.ac.in (P.Y.); 2 Department of Biomedical Engineering, All India Institute of Medical Sciences Delhi, New Delhi 110029, India

**Keywords:** skin tissue, cadaveric skin, simulants, mechanical testing, hyperelasticity

## Abstract

The development of artificial skin that accurately mimics the mechanical properties of human skin is crucial for a wide range of applications, including surgical training for burn injuries, biomechanical testing, and research in sports injuries and ballistics. While traditional materials like gelatin, polydimethylsiloxane (PDMS), and animal skins (such as porcine and bovine skins) have been used for these purposes, they have inherent limitations in replicating the intricate properties of human skin. In this work, we conducted uniaxial tensile tests on freshly obtained cadaveric skin to analyze its mechanical properties under various loading conditions. The stress–strain data obtained from these tests were then replicated using advanced skin simulants. These skin simulants were specifically formulated using a cost-effective and moldable multi-part silicone-based polymer. This material was chosen for its ability to accurately replicate the mechanical behavior of human skin while also addressing ethical considerations and biosafety concerns. In addition, the non-linear mechanical behavior of the developed skin simulants was characterized using three different hyperelastic curve-fit models (i.e., Neo-Hookean, Mooney–Rivlin, and Yeoh models). Moreover, these innovative simulants offer an ethical and practical alternative to cadaveric skin for use in laboratory and clinical settings.

## 1. Introduction

The skin is a layer that protects the human body from the external environment, accounting for approximately 12–15% of total body weight and having an area of around 1.8 m^2^. The thickness of skin varies within the range of 2.1–5.8 mm across different locations of the body [1]. Comprising three layers, the skin consists of the epidermis, the dermis, and the hypodermis. The outermost layer, the epidermis, has a thickness between 60 and 100 μm and serves as a thin, water-resistant, and permeable barrier. The dermis, which lies beneath the epidermis, is 0.6–3 mm thick and provides the skin with its elasticity and strength [2,3]. The hypodermis, also referred to as the subcutaneous layer, is an essential part of the integumentary system, but it is not technically considered a layer of the skin itself. Rather, it serves as the supportive base beneath the skin, comprising loose connective tissue and fat. Its primary functions include cushioning the body, providing insulation, serving as an energy reserve, and facilitating the anchoring of the skin to underlying structures such as muscles and bones, allowing for the skin’s mobility over these areas [4]. Functionally, the skin plays a pivotal role in shielding the body from the external environment. It acts as the first line of defense against all the external pressures encountered throughout life. The skin’s resilience also makes it the initial barrier against physical harm of any kind.

The skin is made up of layers, with the density and arrangement of collagen and elastin fibers determining its stiffness. The skin shows characteristics such as being anisotropic, non-linear, viscoelastic, and almost incompressible [5]. From a mechanical perspective, the skin can exhibit non-linear behavior, with stiffness increasing under higher loads [6,7]. Various methods are used to study the mechanical behavior of skin, including under static and dynamic loading conditions. Researchers have carried out different tests, such as uniaxial, biaxial, and indentation tests, and used imaging techniques to replicate the mechanical properties and biocompatibility of human skin [8,9]. Human tissue simulants are highly desirable and cost-effective in several scenarios, including testing weapons and assessing medical conditions like burns and traumatic injuries [10,11]. Surgeons can use human tissue simulants for robotic surgery training, teaching, and surgical practice. Skin-imitating materials have been developed in the past for examining sports-related injuries, testing ballistic penetration resistance, simulating burnt-skin replacements, and practicing surgical needle insertions [12,13].

Therefore, research on the biomechanical properties of skin tissue is crucial for a number of purposes, such as material modeling of body tissues for surgical repair of skin damage resulting from burns or automotive accidents [14]. In order to determine the mechanical characteristics of pig skin and isotropic silicone rubber at various strain rates, Shergold et al. [15] conducted an experimental investigation. Pig skin stiffens and strengthens with increasing strain rate at a test rate of 0.4 mm/s. Oval-shaped isotropic synthetic skin grafts were studied by Gupta et al. [16], who also looked at the uniaxial tensile properties of isotropic synthetic skin and human skin over a wide range of strain rates. These composite samples behaved mechanically like human skin at a test rate of 0.4 mm/s. Due to several ethical and biosafety concerns, as well as the fact that living tissues are not readily available, studying their mechanical behavior is challenging. It was reported in the literature that human skin and pig skin show similar mechanical properties, but the advantage of synthetic materials is their simplicity and reproducibility [7]. Thus, throughout the last two decades, there have been several efforts to develop human skin simulants [17,18,19]. Silicone surrogates, which mimic the mechanical characteristics of skin tissue and replace cadaveric tissue, are some of the alternative materials utilized for mechanical property investigation of human skin [19]. 

In this work, skin simulants were fabricated using multi-part silicone-based polymeric materials. Cadaveric skin was excised from the thigh regions of fresh cadavers and tested uniaxially to record their stress–strain behavior. Thereafter, candidate skin simulants were fabricated, and the results were compared with the stress–strain plot of cadaveric skin to identify the skin tissue simulants. Section 2 provides a detailed outline of the methodology involved in the sample preparation, their mechanical testing, and non-linear hyperelastic modeling. Section 3 discusses the results of the cadaveric samples, fabricated skin tissue simulants, and hyperelastic curve-fitting coefficients, followed by the conclusions in Section 4.

## 2. Materials and Methods

### 2.1. Sample Preparation from Cadaveric Skin

**Figure 1:**
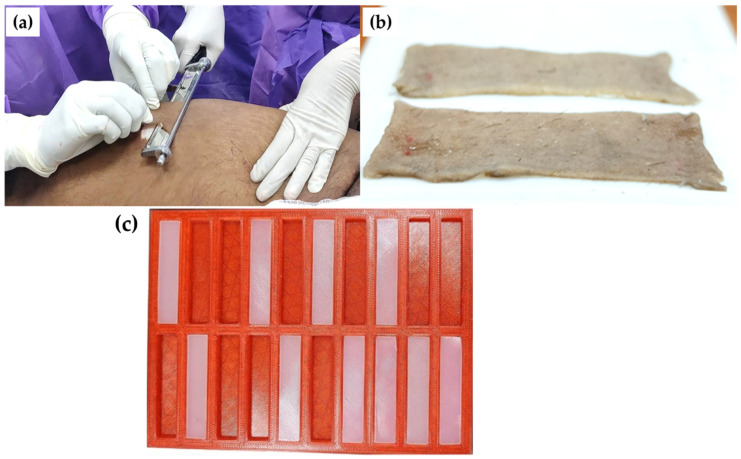
(**a**) Removal of cadaveric thigh skin using dermatone, (**b**) test coupon of cadaveric skin for tensile testing, and (**c**) fabricated test coupons for candidate skin tissue simulants. (**c**) reproduced under license CC-BY-4.0 [21].

Fresh human cadaveric skin was collected from the Department of Forensic Sciences at the All India Institute of Medical Sciences (AIIMS) in Delhi, India (Figure 1). Two cadaveric bodies aged 18 years and 36 years (both male) were used for the sample collection with written consent from the family members of both individuals for the skin collection as per the ethical norms (IEC-138/11.04.2023). From both the cadaveric bodies, samples with a size of 55 × 55 × 1.5 (in mm) were collected from the thigh regions using dermatone. The excised skin samples were stored in a physiological saline solution at a refrigeration temperature of 4 °C to maintain the hydration and properties of the natural skin [20]. Each cadaveric skin sample was divided into five test coupons of size 50 × 10 × 1.5 (in mm) and tested under uniaxial tensile loading within 12 h of the sample collection to maintain the realistic mechanical properties of the human skin.

### 2.2. Designing and 3D Printing of Mold for Simulant Fabrication

The mold for fabricating the skin tissue simulants was designed in SolidWorks 2023 (Dassault Systèmes, Vélizy-Villacoublay, France) with dimensions of 150 × 110 × 5 (in mm). The detailed process and an illustration of mold design can be found in the literature [21]. The mold contained two identical cavities, each measuring 50 × 10 × 1.5 (in mm), intended for casting silicone-based polymeric materials with varying Shore hardness values. The design file was saved in STL format and converted to g-code for 3D printing on a Creality Ender 3 printer from Shenzhen Creality 3D Technology Co., Shenzhen, China, using polylactic acid (PLA) filaments. The printer settings included a printing speed of 45 mm/s, a layer height of 0.1 mm, a nozzle temperature of 210 °C, and a bed temperature of 60 °C. 

Figure 1c illustrates the fabricated test coupons, which were used to mimic the mechanical properties of human skin under uniaxial tensile loading conditions. Silicone-based multi-part polymeric material was procured from Chemzest Enterprises (Chennai, India) with Shore hardness values of 5A, 15A, and 30A for the preparation of test coupons for mechanical testing. The polymer material was cured when part A, containing the hardener, initiated crosslinking upon mixing with part B, resulting in the desired hardness as measured by a Shore durometer.

### 2.3. Fabrication of Candidate Skin Tissue Simulants

The multi-part polymeric material was developed by mixing two different two-part silicone-based polymeric materials with varying Shore hardnesses. A total of 15 test sample coupons (as shown in Table 1) were fabricated to evaluate potential skin tissue simulants, with the composition of the multi-part polymeric materials varying across the samples. The fabricated test coupons had an identical size of 50 × 10 × 1.5 (in mm).

### 2.4. Uniaxial Tensile Testing of Cadaveric Samples and Candidate Skin Tissue Simulants

**Figure 2:**
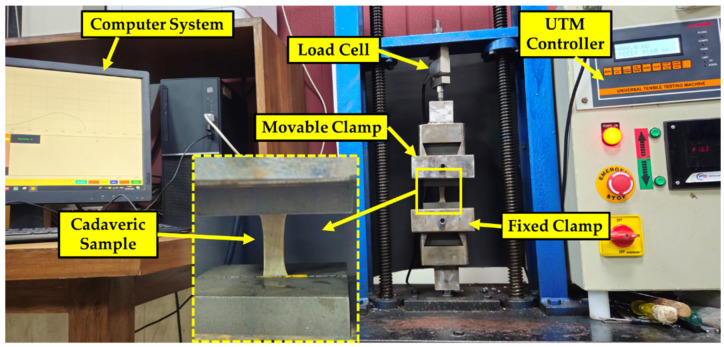
Uniaxial tensile testing of samples to characterize their mechanical properties.

The uniaxial tensile tests of the cadaveric samples and silicone-based polymeric sample coupons, fabricated to simulate human skin, were performed using a universal testing machine (UTM) (Finetechno Engineering Pvt. Ltd., Kolkata, India) with a 100 kg load cell. The samples were clamped securely to prevent slippage during testing and measured 30 mm × 10 mm × 1.5 mm after clamping, with one end fixed and the other pulled. The cadaveric samples and candidate skin tissue simulants were tested at a strain rate of 0.4 mm/s. The specified strain rate was chosen based on studies in the literature that characterized the mechanical properties of cadaveric skin samples from distant body locations [22,23,24,25]. For cadaveric samples, a total of 10 experiments were performed (5 samples from each cadaver), and for the skin tissue simulants a total of 45 experimental runs (15 sample coupons × 3 repetitions) were conducted. The force–displacement data for each experimental run were recorded up to the stretch of 30% of its initial length and converted to stress–strain curves for further comparison. The test procedure involved applying a small pre-load (<0.1 N) to eliminate slack before tensile loading, and each sample was tested three times to ensure repeatability and rule out calibration issues. The testing was carried out at a constant displacement rate of 24 mm/min (0.4 mm/s) to facilitate comparison with existing cadaveric skin studies. The collected data were post-processed to generate engineering stress–strain curves, which involved removing any negative values due to initial slack and shifting the plots to start from the origin. Figure 2 shows the uniaxial tensile testing of the sample on the universal tensile testing machine.

### 2.5. Curve Fitting of the Skin Tissue Simulants Using Hyperelastic Models

Human soft tissues and silicone-based polymeric materials exhibit non-linear mechanical behavior, which is well described by hyperelastic constitutive models such as the Mooney–Rivlin model, the Neo-Hookean model, and the Yeoh hyperelastic curve-fit model [7,26]. A fundamental aspect of hyperelastic material models is the dependence on the strain energy function, denoted as ‘*Ψ*’, and the material type [27]. Moreover, each hyperelastic model depends on the static properties of Cauchy–Green tensors (*I*_1_, *I*_2_, and *I*_3_) or the fundamental stresses (*λ*_1_, *λ*_2_, and *λ*_3_), as seen in Equations (1)–(4). The primary Cauchy stress can be calculated by performing uniaxial tests on any material and according to the methodology outlined in the literature by Martins et al. [26]. Equations (5)–(7) represent the strain energy function (*Ψ*) of the Mooney–Rivlin, Neo-Hookean, and Yeoh hyperelastic curve-fit models used in this work. There exists a non-linear correlation between stress and strain in soft tissues and silicone rubbers.

(1) $$\psi =\psi \left(I_{1},I_{2},I_{3}\right)$$

(2) $$I_{1}=\sum_{i=1}^{3}{\lambda^{2}}_{i}$$

(3) $$I_{2}=\sum_{ij=1}^{3}{\lambda^{2}}_{i}{\lambda^{2}}_{j},i\neq j$$

(4) $$I_{1}=\prod_{i=1}^{3}{\lambda^{2}}_{i}$$

(5) $$\psi_{Mooney-Rivlin}=\sum_{i=1}^{2}c_{i}(I_{1}-3)$$

(6) $$\psi_{Neo-Hookean}=c_{1}(I_{1}-3)$$

(7) $$\psi_{Yeoh}=\sum_{i=1}^{3}c_{i}(I_{1}-3)$$

In Equation (8), the stretch and strain energy functions correspond to the primary Cauchy stress. Equations (5)–(7) for the Mooney–Rivlin, Neo-Hookean, and Yeoh hyperelastic models and Equation (8) for Cauchy stress predict the non-linear characteristics of a sample under uniaxial testing using Equations (9), (10), and (11), respectively.

(8) $$\sigma_{1}=\lambda_{1\;}\frac{\partial \psi }{\partial \lambda_{1\;}}-\lambda_{3}\frac{\partial \psi }{\partial \lambda_{3\;}},\sigma_{2}=\sigma_{3}=0$$

(9) $$\sigma_{Mooney-Rivlin}\;=2(\lambda^{2}-\frac{1}{\lambda })(c_{1}-c_{2}\frac{1}{\lambda })$$

(10) $$\sigma_{Neo-Hookean}\;=2(\lambda^{2}-\frac{1}{\lambda })(c_{1})$$

(11) $$\sigma_{Yeoh}=2(\lambda^{2}-\frac{1}{\lambda })(c_{1}+2c_{2}\;(I_{1}-3)+3c_{3}{\left(I_{1}-3\right)}^{2})$$

The chosen hyperelastic model’s predictive accuracy for the behavior of the skin tissue simulants was evaluated by computing an average R^2^ correlation value, where the R^2^ value varies between 0 and 1, with 0 representing the worst fit and 1 corresponding to the best fit. The values for the Mooney–Rivlin model (*c*_1_ and *c*_2_), the Neo-Hookean model (*c*_1_), and the Yeoh model (*c*_1_, *c*_2_, and *c*_3_) were computed in Section 3.

## 3. Results and Discussion

### 3.1. Stress–Strain Plots of Cadaveric Skin

**Figure 3:**
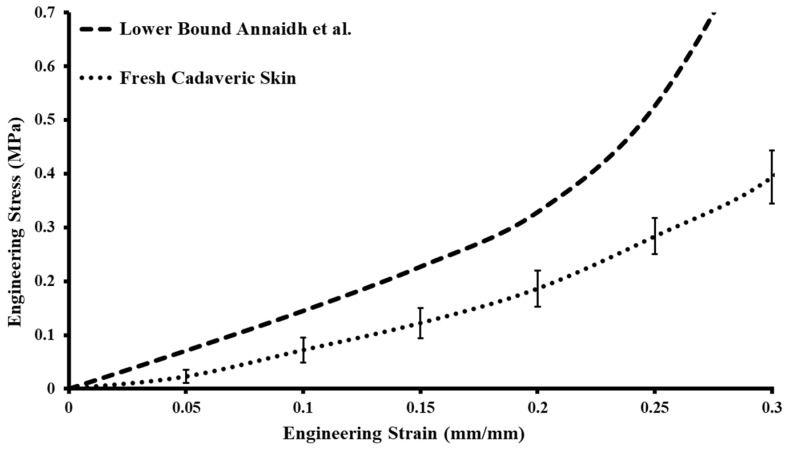
Stress–strain plot of fresh cadaveric skin compared with the lower bound from the literature [28].

The uniaxial tensile tests were conducted on six cadaveric samples, which were collected from the thigh regions of two cadavers, as discussed in Section 2. The tests were performed with a sample size of 50 × 10 × 1.5 (in mm) at a strain rate of 0.4 mm/s and up to a stretch of 30%. The stress value was calculated by dividing the applied force by the cross-sectional area of the specimen, and the strain was calculated by dividing the change in length of the specimen from its original size. Figure 3 illustrates the stress–strain results of the tested samples, with error bars indicating the variation in stress values across all the cadaveric sample tests. The deviation bar represents the upper and lower bounds of the skin samples tested in our study. The stress–strain plots revealed that the tested cadaveric samples aligned with studies in the literature, validating the stress–strain values of the tested cadaveric samples [28]. The maximum stress value recorded for the cadaveric skin samples was 0.393 MPa, with a standard deviation of ±0.049 MPa. In the literature, Annaidh et al. [28] used cadaveric skin from different locations of a cadaver and reported the upper and lower bounds of the human skin. The stress–strain plot of the present study showed comparable results and was considered for the development of skin tissue simulants (Figure 3).

### 3.2. Stress–Strain Plots of the Developed Skin Tissue Simulants

**Figure 4:**
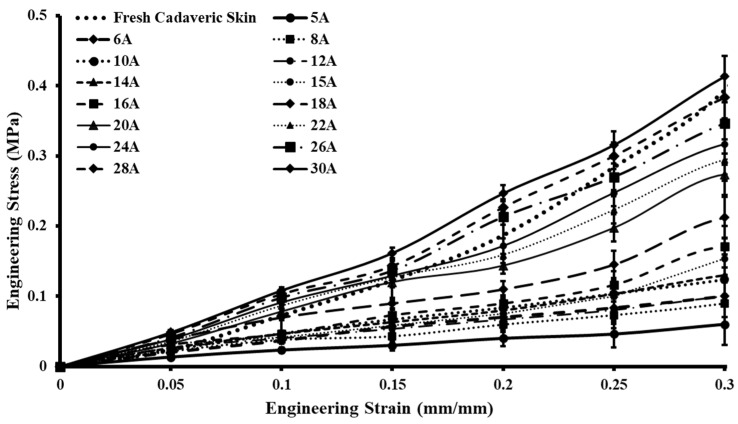
Stress–strain plot of uniaxially tested candidate skin tissue simulants compared with the stress–strain results of fresh cadaveric skin.

The uniaxial tensile tests were performed on 15 skin simulant samples with varying Shore hardnesses (5A–30A) at a strain rate of 0.4 mm/s (24 mm/min), which was similar to uniaxial testing on fresh cadaveric human skin. The plots of the tested samples for the skin tissue simulants were compared with the cadaveric samples of fresh skin tested. The stress vs. strain graphs were created utilizing the average stress values of each test sample (i.e., 5A–30A) coupled with the standard deviation error bars (Figure 4). To ensure the reliability of the tensile tests, three samples of each Shore hardness (5A–30A) were fabricated and tested. It was found that the highest stress values at 30% strain for 30A simulants were 0.413 ± 0.0295 MPa, and the lowest value was recorded for the 5A simulants, i.e., 0.06 ± 0.007 MPa. The simulants with Shore hardnesses of 5A–24A were found under the bound of fresh cadaveric skin (Figure 4), and the simulants with Shore hardnesses of 20A, 22A, and 24A closely mimicked the stress–strain plot of fresh cadaveric skin. It should be mentioned that the silicone-based polymeric composition of the simulant samples did not break in the same way as that of actual human skin. However, it did show ultimate tensile stress values similar to those of human skin [28]. The study revealed that stress values were directly associated with the Shore hardness of the developed test coupon, i.e., as the Shore hardness increases, so does the stress value.

### 3.3. Repeatability Test of the Controlled Samples

**Figure 5:**
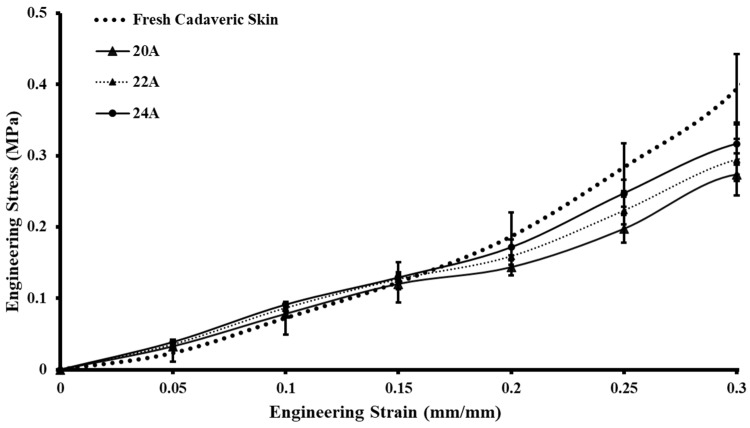
Controlled skin tissue simulants mimicking the stress–strain results of the fresh cadaveric skin.

The candidate samples with Shore hardnesses of 20A, 22A, and 24A closely mimicked the stress–strain behavior of fresh cadaveric skin. From the stress-versus-strain plot (see Figure 5), these simulant samples were identified as the controlled test samples for the repeatability test. All these controlled samples were fabricated using silicone-based polymeric material of two different Shore hardnesses (i.e., 15A and 30A). For the first controlled sample, i.e., 20A, the multi-part composition of the silicone-based polymeric material was 33% part A and 33% part B of the 15A Shore hardness silicone-based polymeric material and 17% part A and 17% part B of the 30A Shore hardness silicone-based polymeric material. For the second controlled sample (22A), the silicone-based polymeric material consisted of 27% part A and 27% part B of the 15A Shore hardness polymeric material and 23% part A and 23% part B of the 30A Shore hardness polymeric material. For the third controlled sample, denoted as 24A, the silicone-based polymeric material was composed of 20% part A and 20% part B of the silicone-based polymeric material with a Shore hardness of 15A and 30% part A and 30% part B of the silicone-based polymeric material with a Shore hardness of 30A. To confirm the reproducibility of the results, three sets of uniaxial tests were conducted three times for all three controlled samples, and the simulants were able to replicate the same results. It is important to clarify that the controlled specimens were labeled to compare the experimental results with those recorded for the fresh cadaveric skin.

### 3.4. Hyperelastic Curve-Fitting Modeling of Skin Tissue Simulants

The stress-versus-strain response of the test coupons for the candidate skin tissue simulants was accurately characterized using non-linear hyperelastic curve-fit models. The stress–strain behavior of the multi-part silicone-based polymeric samples (10A–30A) was quantified and characterized using Mooney–Rivlin, Yeoh, and Neo-Hookean curve-fitting coefficients (Equations (8), (9), and (10), respectively). The experimental stress values were curve-fitted using these models, and the coefficients (*c*_1_, *c*_2_, and *c*_3_) are listed in Table 2. The accuracy of predicting the non-linear behavior of skin tissue simulants was assessed for each hyperelastic model (Table 2) by numerically estimating the average R^2^ correlation for each sample, which ranged between 0.9 and 1. The R^2^ correlation value was 0.94 for the Mooney–Rivlin model, 0.98 for the Yeoh Model, and 0.91 for the Neo-Hookean hyperelastic model.

## 4. Conclusions

In this study, we focused on developing and testing skin tissue simulants using multi-part silicone-based polymeric materials with varying Shore hardnesses. The goal was to accurately mimic the mechanical properties of human skin for various applications. To evaluate the stress-versus-strain behavior of the skin tissue simulants, we created a total of 15 compositions by mixing the multi-part silicone-based polymeric materials, as outlined in Table 1. We then compared the stress-versus-strain behavior of the fabricated samples with cadaveric skin samples to identify the material composition that best replicated the mechanical properties of natural skin. After extensive testing, we identified compositions that successfully simulated the mechanical properties of skin tissue and selected three controlled samples for further testing to ensure repeatability and consistency. 

The developed skin tissue simulants have various translational impacts, being usable for medical applications, crash testing, and ballistics testing. Skin tissue simulants with realistic mechanical properties have significant applications in medical device testing, enabling accurate evaluation of wound care products, surgical tools, and wearable sensors. In training and education, they provide a realistic medium for practicing surgical techniques and emergency response procedures. For research, these simulants are anticipated to be used in the study of injury mechanics, drug delivery systems, and skin–device interactions. Additionally, they play a crucial role in cosmetic product testing and serve as ethical alternatives to animal testing in regulatory science.

While our approach yielded valuable insights, it is important to acknowledge certain limitations in the framework of the multi-part silicone-based polymer material system. First, skin tissue simulants were developed using an isotropic material, whereas human skin exhibits anisotropic behavior due to the presence of Langer lines. Second, future studies need to consider biaxial tensile testing to achieve more realistic results. Third, it is important to note that the pre-stress and load responses of natural skin may vary with factors such as ageing, humidity, and temperature, none of which were replicated by the fabricated silicone-based polymeric skin tissue. Fourth, in the current approach, we only modeled a single dermis layer of the skin, whereas, in reality, skin consists of underlying tissues, fat, and connective tissues, all of which influence its mechanical properties. In the future, composite-based anisotropic skin tissue surrogates can be fabricated to more accurately simulate the mechanical properties of multi-layered skin. Fifth, biological and biochemical properties, such as compatibility and permeability, can be incorporated using advanced material modeling techniques to develop high-fidelity skin simulants for a wider range of applications.

## Figures and Tables

**Table 1:** Mixing ratio of multi-part silicone-based polymeric materials (by weight %).

Sr. No.	Shore Hardness	Material of Shore 5A		Material of Shore 15A		Material of Shore 30A	
		Part A	Part B	Part A	Part B	Part A	Part B
1	5A	50	50	–	–	–	–
2	6A	45	45	5	5	–	–
3	8A	35	35	15	15	–	–
4	10A	25	25	25	25	–	–
5	12A	15	15	35	35	–	–
6	14A	5	5	45	45	–	–
7	15A	–	–	50	50	–	–
8	16A	–	–	47	47	3	3
9	18A	–	–	40	40	10	10
10	20A	–	–	33	33	17	17
11	22A	–	–	27	27	23	23
12	24A	–	–	20	20	30	30
13	26A	–	–	13	13	37	37
14	28A	–	–	7	7	43	43
15	30A	–	–	–	–	50	50

**Table 2:** Hyperelastic curve-fit coefficients of fabricated samples for skin tissue surrogates.

Sr. No.	Shore Hardness of Sample	Mooney–Rivlin		Yeoh			Neo-Hookean
		*c*_1_	*c*_2_	*c*_1_	*c*_2_	*c*_3_	*c*_1_
1	5A	0.0096019	0.0006	0.00380	0.000594	0.000004	0.0019406
2	6A	0.0132332	0.0011	0.00941	0.000094	0.000011	0.0055783
3	8A	0.0142083	0.0013	0.00970	0.000114	0.000023	0.0066360
4	10A	0.0164332	0.0014	0.01080	0.000894	0.000029	0.0108246
5	12A	0.0171486	0.0020	0.01233	0.000002	0.000034	0.0057830
6	14A	0.0173259	0.0030	0.01301	0.000004	0.000042	0.0084725
7	15A	0.0175032	0.0035	0.01358	0.000012	0.00005	0.0094663
8	16A	0.0188794	0.0038	0.01365	0.000015	0.000059	0.0104602
9	18A	0.0292954	0.0047	0.02251	0.000023	0.000078	0.0213964
10	20A	0.0328677	0.0058	0.02566	0.000027	0.000084	0.0239112
11	22A	0.0384312	0.0063	0.02849	0.000031	0.000119	0.0321826
12	24A	0.0428568	0.0071	0.03171	0.000033	0.000134	0.0334261
13	26A	0.0523601	0.0084	0.04110	0.000038	0.000135	0.0403316
14	28A	0.0591392	0.0089	0.04334	0.000048	0.000192	0.0488824
15	30A	0.0661843	0.0112	0.04938	0.000061	0.000205	0.0538254

## Data Availability

The datasets generated during and/or analyzed during the current study are not publicly available due to their size but are available from the corresponding author on reasonable request.
